# Low‐Voltage Oscillatory Neurons for Memristor‐Based Neuromorphic Systems

**DOI:** 10.1002/gch2.201900015

**Published:** 2019-08-07

**Authors:** Qilin Hua, Huaqiang Wu, Bin Gao, Qingtian Zhang, Wei Wu, Yujia Li, Xiaohu Wang, Weiguo Hu, He Qian

**Affiliations:** ^1^ Institute of Microelectronics Tsinghua University Beijing 100084 China; ^2^ CAS Center for Excellence in Nanoscience Beijing Key Laboratory of Micro‐nano Energy and Sensor Beijing Institute of Nanoenergy and Nanosystems Chinese Academy of Sciences Beijing 100083 China; ^3^ School of Microelectronics Tianjin University Tianjin 300072 China

**Keywords:** memristors, neuromorphic, oscillatory neurons, spiking neural networks, threshold switching

## Abstract

Neuromorphic systems consisting of artificial neurons and synapses can process complex information with high efficiency to overcome the bottleneck of von Neumann architecture. Artificial neurons are essentially required to possess functions such as leaky integrate‐and‐fire and output spike. However, previous reported artificial neurons typically have high operation voltage and large leakage current, leading to significant power consumption, which is contrary to the energy‐efficient biological model. Here, an oscillatory neuron based on Ag filamentary threshold switching memristor (TS) that has a low operation voltage (<0.6 V) with ultralow power consumption (<1.8 µW) is presented. It can trigger neuronal functions, including leaky integrate‐and‐fire and threshold‐driven spiking output, with high endurance (>10^8^ cycles). Being connected to an external resistor or a resistive switching memristor (RS) as synaptic weight, the TS clearly demonstrates self‐oscillation behavior once the input pulse voltage exceeds the threshold voltage. Meanwhile, the oscillation frequency is proportional to the input pulse voltage and the conductance of RS synapse, which can be used to integrate the weighted sum current. As an energy‐efficient memristor‐based spiking neural network, this combination of TS oscillatory neuron with RS synapse is further evaluated for image recognition achieving an accuracy of 79.2 ± 2.4% for CIFAR‐10 subset.

Neuromorphic systems, which can leverage the distributed computing in neurons and localized storage in synapses, is a promising building block to overcome the bottleneck of von Neumann architecture.^1^, ^2^, ^3^, ^4^, ^5^, ^6^ To date, artificial synapses have been successfully realized by using resistive switching memristors^7^, ^8^, ^9^ (RS) to emulate biosynaptic functions including spike‐time‐dependent plasticity (STDP),^7^ pair‐pulse facilitation/depression (PPF/PPD),^8^ and long‐term potentiation/depression (LTP/LTD).^9^ On the other hand, artificial neurons are typically based on complex CMOS circuits^10^ with several active components to implement neuronal functions, which suffer from low energy efficiency and limit integration density.^11^ Recently, threshold switching memristors (TS), typically with large on/off ratio, simple structure, and scaling capability, have tremendous potentials in applications such as selectors for nonvolatile data storage,^12^, ^13^ steep slope transistors for logic devices,^14^, ^15^ and neurons for neuromorphic computing.^16^, ^17^, ^18^ Furthermore, by not using inductive components or transistors, TS based neuron devices are capable of scaling down and increase the integration density.^11^ In addition, an applied voltage on the TS device will induce self‐oscillation behavior due to the *I*–*V* hysteresis in TS.^11^, ^19^, ^20^ And oscillation parameters such as frequency or phase can be used to store the input information from voltage or conductance.^2^, ^20^ In fact, oscillatory neurons are typically coupled to a resistive memory (i.e., RS) to form neural networks, which is very promising for implementing neuromorphic systems due to rapid recognition speed and low power consumption.^2^, ^21^

Recently, various TS devices based on different materials, such as insulator–metal‐transition (IMT, e.g., NbO_2_ 16 and VO_2_ 22), chalcogenide (e.g., GeSe^23^), and transition metal oxide (e.g., TaO*_x_* 21), were reported to show the oscillation behavior for implementing neuromorphic applications. However, these TS devices typically withstand high operation voltage (e.g., Ge_2_Sb_2_Te_5_ RESET: ≈5.3 V^24^) and large leakage current (e.g., NbO_2_: >10 µA^11^), and thus require more power supply, as shown in Table S1 (Supporting Information). It is worth noting that such a high working voltage will seriously affect the resistance state in most RS devices, and hence low voltage oscillatory neurons are highly desired for novel neuromorphic systems.

Currently, metal filamentary TS devices, consisting of Ag (or Cu) as active electrode or dopant in solid electrolyte, have been demonstrated on low threshold voltage and low leakage current characteristics.^25^ However, a volatile threshold switch would typically transit to nonvolatile resistive switch after continuous cycling, as a result of stable filament growth.^26^ It should be noted that a highly reliable metal filamentary TS with low threshold voltage and reduced off‐state leakage is much needed that can contribute to obtain a wider weight range for oscillatory neuronal functions. Here, we present an oscillatory neuron based on Ag filamentary TS that has a low operation voltage (<0.6 V) with ultralow power (<1.8 µW) and highly reliable on/off switching (>10^8^ cycles). And we demonstrate the capability of memristor‐based spiking neural network for image recognition with an accuracy of 79.2 ± 2.4% for CIFAR‐10 subset.

**Figure** **1**a schematically illustrates a biological neural system model consists of neurons and synapses to process information with the energy‐efficient paradigm. The biological neuron is essentially capable of integrating inputs from preneurons via synapses, and triggers an output action potential when the membrane potential reaches a threshold level. As inspired by the biological model, the equivalent electronic model with artificial neurons and synapses is proposed to implement the functions of spiking neural network (Figure 1b). More specifically, the artificial TS neuron can be used to accumulate input signals from preneurons through RS synapses, and generate output spikes in response to decide whether the signals transmit or not. In addition, the operation voltages of the TS neuron and the RS synapse in neuromorphic systems should be evaluated simultaneously. If the input pulse voltage (*V*_pulse_) is close to the read pulse voltage (*V*_read_) of the RS synapse, the TS neuron will trigger to oscillate and the neuromorphic system can work normally. However, if *V*_pulse_ is close to the write pulse voltage (*V*_write_) of the RS synapse, the neuromorphic system will do not work, as a result of high voltage seriously affecting resistance state of the RS synapse (see Figure S1 in the Supporting Information). Thus, it is highly desirable to present a reliable TS device with small voltage and low leakage current for practical oscillatory neuron applications.

**Figure 1:**
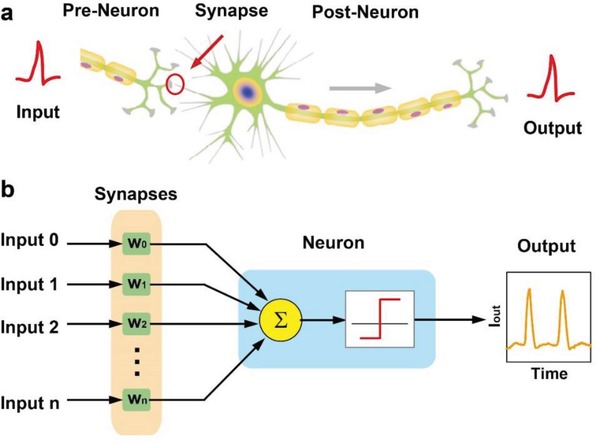
Schematic illustration of neuromorphic system. a) Biological model: the biological neuron receives inputs from other neurons by interconnected synapses. b) Equivalent electronic model: the electronic neuron for accumulating inputs generated by different preneurons through resistive switching memristor (RS) synapses to implement the functions of spiking neural network.

Recent reports show that the Ag filamentary TS can meet the requirements both for small operation voltage and low leakage current. **Figure** **2**a shows the as‐fabricated TS with Pt/Ag nanodots/HfO_2_/Pt stacks, according to cross‐sectional scanning transmission electron microscopy (STEM) image. For the TS device, we introduced Ag nanodots as electrochemically active electrode at the interface of HfO_2_ layer and Pt top electrode. Such active electrode configuration could avoid excessive Ag atoms migration into HfO_2_ during operations, and with rapid thermal processing (RTP) would contribute to the formation and rupture of multiple weak Ag conductive filaments (CFs). The surface morphology of Ag nanodots without or with RTP treatment is characterized by using an atomic force microscopy (AFM), as shown in Figure S2 (Supporting Information). Both Ag thin layers (<4 nm) are not continuous, and the RTP‐treated one shows more obvious separation state of Ag nanodots. Moreover, the RTP treatment could make some Ag atoms prediffused into the HfO_2_ electrolyte, which would accumulate on the interface of bottom electrode/HfO_2_. Figure 2b illustrates the energy‐dispersive X‐ray spectroscopy (EDS) line profiles of the TS stacks, indicating that Ag distributes at both interfaces of HfO_2_ after cyclic *I*–*V* tests.

**Figure 2:**
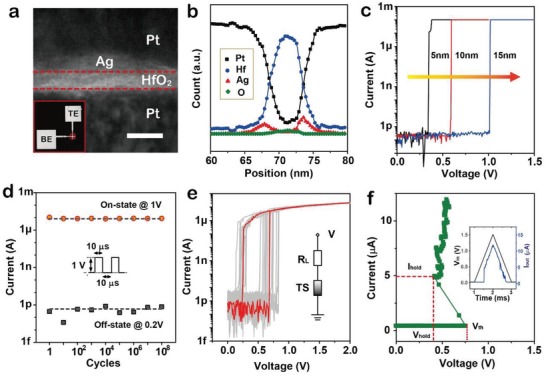
Characterizations of Ag filamentary threshold switching memristor (TS). a) Cross‐sectional TEM image of the proposed TS stacks. b) Energy‐dispersive X‐ray spectroscopy (EDS) line profiles of the TS stacks. c) *I*–*V* characteristics of TS at different thickness of HfO_2_ layer (5, 10, and 15 nm), indicating threshold voltage (*V*_th_) can be controlled with HfO_2_ thickness. d) Reliable analysis (endurance test) indicates superior on/off switching over 10^8^ cycles with connecting a series resistor of 81 kΩ. Applying 10 µs, 0.5 V pulse; time interval: 20 µs; read voltages: 0.1 V/1 V. e) DC *I*–*V* characteristics of TS connected with a load resistor (*R*_L_ = 81 kΩ) in 20 devices. f) AC *I*–*V* characteristic of TS connected with *R*_L_ = 81 kΩ, which is measured by applying a triangle voltage pulse (1.5 V, 2 ms).

In Figure S3a (Supporting Information), the conventional Ag filamentary TS devices would lead to nonvolatile memory switching transition when compliance current (*I*_cc_) > 10 µA or undergoing continuous cycling, as a result of large/stable filament growth (Figure S3b, Supporting Information). On the contrary, the presented TS devices, which still maintains the volatile threshold switching behavior even at *I*_cc_ > 100 µA, can represent improved on‐state current, extremely high on/off ratio > 10^9^, and ultralow leakage current < 1 pA, as shown in Figure S3c (Supporting Information). The performance comparison of various TS devices are also summarized in Table S2 (Supporting Information). Through RTP treatment, the pre‐diffused Ag atoms in HfO_2_ layer contribute to induce multiple channels that generate larger intensities in electric field along the Ag nanoparticle directions. Figure S3d (Supporting Information) schematically illustrates working mechanism of Ag filamentary TS that is the formation and rupture of multiple weak CFs. Upon the applied voltage exceeds a threshold voltage (*V*_th_), CFs would be formed to bridge the top and bottom electrodes, enabling the TS switching to on‐state. Upon the applied voltage reduced below hold voltage (*V*_hold_), CFs come to spontaneous rupture due to the Gibbs–Thomson^26^ or nanobattery effect,^27^ leading to TS finally transition to off‐state. Remarkably, the *V*_th_ can be reduced from 1 to 0.3 V by shrinking the thickness of HfO_2_ layer from 15 to 5 nm (Figure 2c), which could provide the possibility to scaling voltage to sub 0.3 V for TS oscillatory neuron. In addition, a highly reliable on/off switching characteristic (that is, endurance > 10^8^ cycles) is clearly shown in Figure 2d, and it represents that the TS devices could ensure long‐term stable operations in repeated oscillations.

The TS device with a crosspoint stack area of 2  ×  2 *µ*m^2^ is used to implement oscillatory neuronal functions. By serially connection with a suitable load resistor (*R*_L_), the TS device is able to demonstrate self‐oscillation behavior. Figure 2e shows DC *I*–*V* characteristics of the TS device from device‐to‐device when connected with a *R*_L_ of 81 kΩ. It indicates the highly reliable switching of the TS devices. Moreover, the negative differential resistance (NDR) effect is clearly observed in Figure 2f, by applying a triangle pulse voltage sweep (1.5 V, 2 ms). When the applied voltage is close to *V*_th_, the resistance of the TS device will transit from the off‐state resistance (*R*_off_) to the on‐state resistance (*R*_on_), and the voltage stressing on the TS device will fast reduce to *V*_hold_, which ultimately leads to an increase in current. Obviously, the TS device is not capable of oscillating when applying an input pulse voltage of 0.5 V (in **Figure** **3**a), due to not reach the threshold level yet. Furthermore, the self‐oscillation characteristics of the TS device at different input pulse voltages (0.6, 0.7, and 0.8 V) can be found in Figure 3b–d, which is as a result of spontaneous formation and rupture of CFs. In addition, the TS oscillatory neuron shows time delay before its oscillation. The delay time may correspond to the tolerance of TS for voltage stress, so that the TS device finally turns on under voltage bias. Besides, the delay time indicates the integration functionality of the TS oscillatory neuron, and it will be further reduced with a larger input voltage pulse.

**Figure 3:**
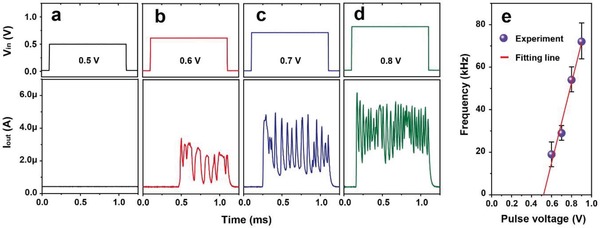
Dynamics of TS oscillatory neuron. a–d) Oscillation current responds to input pulse of 0.5, 0.6, 0.7, and 0.8 V, respectively. TS is in series with *R*_L_ = 81 kΩ. Self‐oscillation behavior is observed when input pulse voltage over a threshold value. e) Oscillation frequency as a function of pulse voltage, and there is a threshold of pulse voltage (≈0.52 V) to oscillate in the TS oscillatory neuron. The frequency is derived from the oscillation current by using fast Fourier transformation (FFT), whose plots are shown in Figure S4 (Supporting Information).

The *R*_L_ should be between *R*_on_ and *R*_off_ to conduct oscillation, and there is a parasitic capacitance at the TS device.^11^, ^20^ When applying an input pulse voltage, the voltage drops on the TS device (*R*_off_ > *R*_L_), and the parasitic capacitance comes to be charged. When the voltage is over *V*_th_, the resistance of the TS device transits to *R*_on_, and the voltage mainly drops on the *R*_L_ (*R*_on_ < *R*_L_), leading to the discharging of the parasitic capacitance. Once the voltage drops on the TS device below *V*_hold_, the resistance of the TS device returns to *R*_off_. In fact, the oscillation is generated with spontaneous voltage dividing on the resistance of TS (*R*_TS_: *R*_off_ → *R*_on_ → *R*_off_). As it described above, the TS device would work in oscillation between *V*_th_ and *V*_hold_ with a suitable *R*_L_ connection.

Moreover, the oscillation frequency can be derived from the oscillation current by using fast Fourier transformation (FFT). And the corresponding FFT plots (from Figure 3e) that identify the frequency peaks are shown in Figure S4 (Supporting Information). The oscillation frequency has a marked increase from 19 to 54 kHz when the input voltage pulse increases from 0.6 to 0.8 V, as shown in Figure 3b–d. Similarly, the oscillation frequency will increase as the reduction of *R*_L_ (corresponding to the weight of RS synapse), i.e., 72 kHz for *R*_L_ = 81 kΩ and 59 kHz for *R*_L_ = 100 kΩ (Figure S5, Supporting Information). Figure 3e shows the TS oscillatory neuron that is only observed to trigger oscillation characteristics when the input pulse voltage is above a critical threshold. Obviously, the oscillation frequency is proportional to the input voltage pulse, and the threshold that starts to oscillate is ≈0.52 V, which is derived from fitting the linear relation between the oscillation frequency and the input pulse voltage.

In fact, Ag filamentary threshold switching memristors inherently have significant variations from cycle‐to‐cycle or device‐to‐device, due to the stochastic dynamics of filament growth.^28^ Consequently, the controllability, stability, and repeatability of the oscillations should be improved to satisfy with the practical applications of the neuromorphic systems involving multiple neurons. Recently, some strategies have been proposed to reduce the device variations, e.g., dielectrics engineering by orienting grain boundaries,^29^ filament confining in a small region by using a nanowire dielectric,^30^ Ag nanowires embedded in a polymer,^31^ graphene defects,^32^ or highly ordered Ag nanodots.^33^

Based on the threshold‐driven oscillation behavior of the low voltage TS, a neuromorphic system is proposed to exploit the RS synapse and the TS oscillatory neuron, demonstrating the capability of memristor‐based spiking neural network for image recognition. Herein, the TaO*_x_*/HfO_2_ based RS array cells are employed as electronic synapses, and the TS oscillatory cells are employed as leaky integrate‐and‐fire neurons. Figure S6 (Supporting Information) illustrates the typical conductance training of analog‐like RS electronic synapse, and is demonstrated with linear analog SET and RESET, 50 ns speed, 10 × analog tuning window, and 100 kΩ on‐state resistance for multilevel states.

A subset of grayscale CIFAR‐10 is used to demonstrate the image recognition application, as shown in Figure S7 (Supporting Information). Five classes of images, airplane/automobile/dog/horse/ship, are chosen for the task. The architecture of the memristor‐based spiking neural network is illustrated in **Figure** **4**a and Figure S8 (Supporting Information). Indeed, the oscillation frequency, which is proportional to the input pulse voltage, represents a weighted sum when the TS oscillatory neuron connected to all the RS synaptic weights in one column of the array. The stochastic gradient decent with backpropagation is used to train the networks. The weight decay is 0.0001, the momentum is 0.9, and the minibatch size is 200. The weights are initialized as in ref. 34. The batch normalization^35^ is used. The initial learning rate is 0.5 and is divided by 10 at 5 and 8 epochs. The training is terminated at 10 epochs, which is determined on a 20k/5k train/validation split. For test, all the 5k test images from the five selected classes are used. And an average recognition accuracy of 79.2 ± 2.4% is achieved, as shown in Figure 4b.

**Figure 4:**
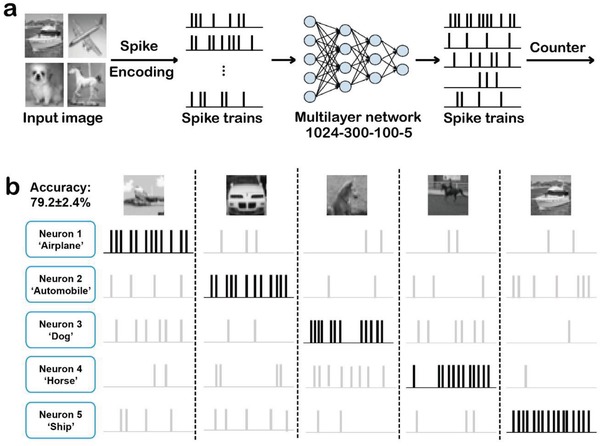
Simulated neuromorphic system for image recognition. a) Illustration for memristor‐based spiking neural networks system. Use Siegert transformation module to transform each pixel into a spike train.^36^ b) Recognition results of memristor‐based spiking neural networks system for CIFAR‐10 subset.

In summary, Ag filamentary TS device achieves a lower voltage of 0.6 V, much reduced power consumption of ≈1.8 µW, and highly reliable switching characteristics (see Table S1 in the Supporting Information), which is very suitable as an oscillatory neuron in neuromorphic systems. The oscillation frequency of the TS is proportional to the input pulse voltage or the synaptic weight, and has a threshold of ≈0.52 V for self‐oscillation. In the neuromorphic system consisting of RS synapse and TS oscillatory neuron, we demonstrate the energy‐efficient memristor‐based spiking neural network that achieves an accuracy of 79.2  ±  2.4% in image recognition for CIFAR‐10 subset. Looking forward, the low voltage TS oscillatory neuron has a very promising potential in novel neuromorphic system applications.

## Experimental Section

*Fabrication of Ag Filamentary Threshold Switching Memristor*: The device stacks were prepared on SiO_2_/Si wafer substrate. The bottom electrode was patterned by photolithography (Cannon PLA 550), sputtered (Kurt J. Lesker LAB18) with 5 nm Ti and 50 nm Pt, and then lift‐off the thin films. HfO*_x_* thin films (5, 10, and 15 nm) were prepared by atomic layer deposition (ALD, Beneq TFT 200) at 200 °C. Four nanometer Ag thin films were deposited by E‐beam evaporator (Denton, explorer 128) and followed by the RTP at 500 °C for 30 s. Afterward, 40 nm Pt thin film as the top electrode, patterned and deposited by lithography and sputter, respectively.

*Microstructural Characterizations*: TEM cross‐sectional samples were prepared using FIB (FEI Helios). STEM image and EDS line profile were obtained by STEM (FEI Tecnai F20).

*Electrical Measurements*: DC *I*–*V* characteristics were measured by Agilent B1500A semiconductor device parameter analyzer. AC *I*–*V* characteristics and oscillation behavior were measured by Agilent B1530A Waveform Generator/Fast Measurement Unit (WGFM) and Agilent B1500A semiconductor device parameter analyzer. Endurance measurements were conducted by a test system consisting of Agilent B1110A pulse‐/pattern generator, Agilent B1500A semiconductor device parameter analyzer and Agilent B2201A 14ch low leakage switch mainframe.

## Conflict of Interest

The authors declare no conflict of interest.

## Supporting information

Supplementary
