# Supplementary material for: Low‐Voltage Oscillatory Neurons for Memristor‐Based Neuromorphic Systems
Source: Glob Chall. 2019 Aug 7;3(11):1900015. doi: 10.1002/gch2.201900015 (PMC6827597; doi:10.1002/gch2.201900015)
Supplement: Supplementary file 1 — Supplementary [file GCH2-3-1900015-s001.pdf]

Copyright WILEY-VCH Verlag GmbH & Co. KGaA, 69469 Weinheim, Germany, 2019.

Global Challenges
Open Access

Supporting Information

for *Global Challenges,* DOI: 10.1002/gch2.201900015

Low-Voltage Oscillatory Neurons for Memristor-Based Neuromorphic Systems

Qilin Hua, Huaqiang Wu,* Bin Gao,* Qingtian Zhang, Wei Wu, Yujia Li, Xiaohu Wang, Weiguo Hu, and He Qian*

Copyright WILEY-VCH Verlag GmbH & Co. KGaA, 69469 Weinheim, Germany, 2016.

Supporting Information

Low Voltage Oscillatory Neurons for Memristor-based Neuromorphic Systems

Qilin Hua, Huaqiang Wu*, Bin Gao*, Qingtian Zhang, Wei Wu, Yujia Li, Xiaohu Wang, Weiguo Hu, He Qian*

Dr. Q. Hua, Prof. H. Wu, Prof. B. Gao, Dr. Q. Zhang, Dr. Wei Wu, Y. Li and Prof. H. Qian
Institute of Microelectronics, Tsinghua University, Beijing, 100084, China
E-mail: wuhq@tsinghua.edu.cn; gaob1@ tsinghua.edu.cn

Dr. Q. Hua, Prof. W. Hu
CAS Center for Excellence in Nanoscience, Beijing Key Laboratory of Micro-nano Energy and Sensor, Beijing Institute of Nanoenergy and Nanosystems, Chinese Academy of Sciences, Beijing, 100083, China
E-mail: huaqilin@binn.cas.cn

Dr. X. Wang
School of Microelectronics, Tianjin University, Tianjin, 300072, China

Keywords: threshold switching, memristor, oscillatory neuron, neuromorphic, spiking neural network

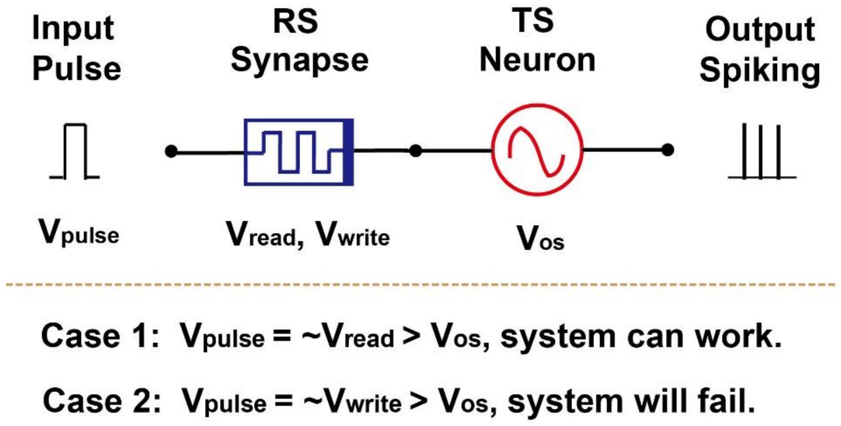

Figure S1. Illustration of memristor-based neuromorphic system consisting of RS synapse and TS neuron to generate output spike. Low voltage is necessary for TS oscillatory neuron to be capable of operating the system well.

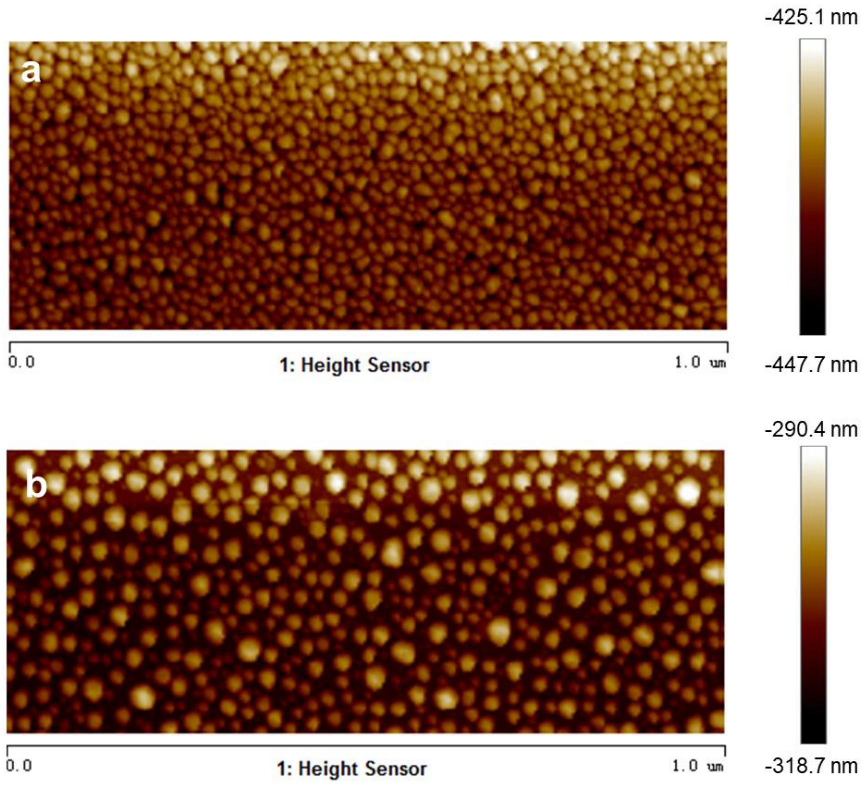

Figure S2. Surface morphology of Ag nanodots deposited on HfO_2 thin film (a) without and (b) with rapid thermal processing (RTP). Both Ag thin layers (< 4 nm) are not continuous, and the RTP-treated one shows more obvious separation state of Ag nanodots.

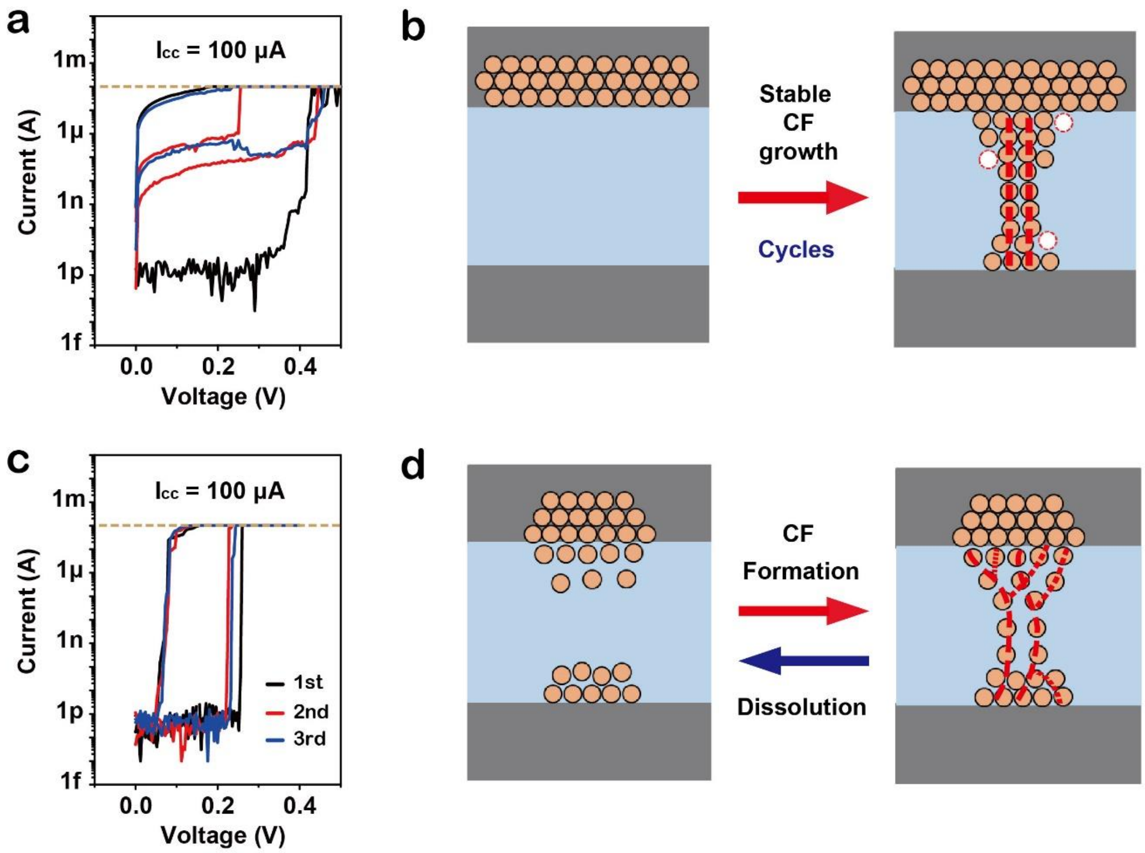

Figure S3. I-V characteristics of TS and the mechanism. (a) The TS device without optimization shows non-volatile resistance transition to some extent during the cyclic I-V sweeping under large compliance current (I_{cc} = 100 μA). (b) Schematic illustration for stable conductive filament (CF) growth in cycles. (c) The TS device with optimization (rapid thermal processing, RTP) shows volatile threshold switching during the cyclic I-V sweeping under I_{cc} of 100 μA. (d) Schematic illustration for the formation and dissolution of multiple weak CFs to conduct highly reliable threshold switching behavior.

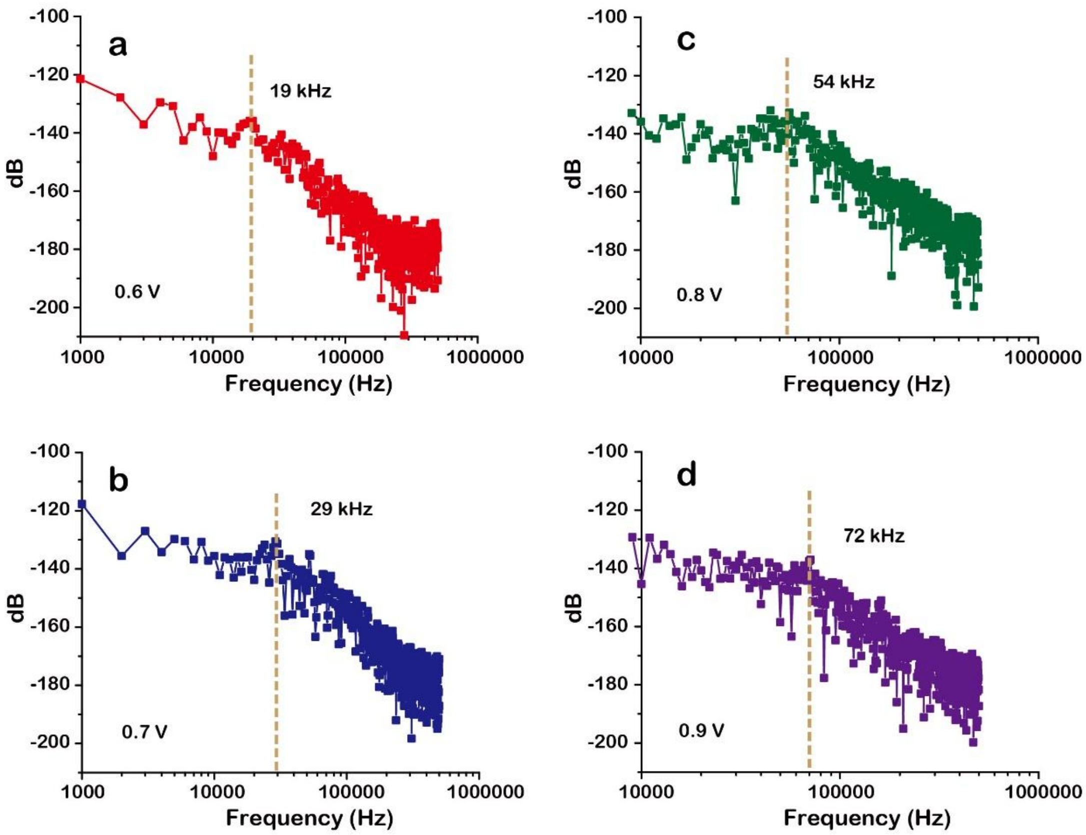

Figure S4. Fast Fourier Transform (FFT) plots of the TS oscillatory neuron at different input voltage pulse of (a) 0.6 V, (b) 0.7 V, (c) 0.8 V, and (d) 0.9V, as depicted in Figure 3e. The frequency peaks are clearly identified as shown with the dashed line.

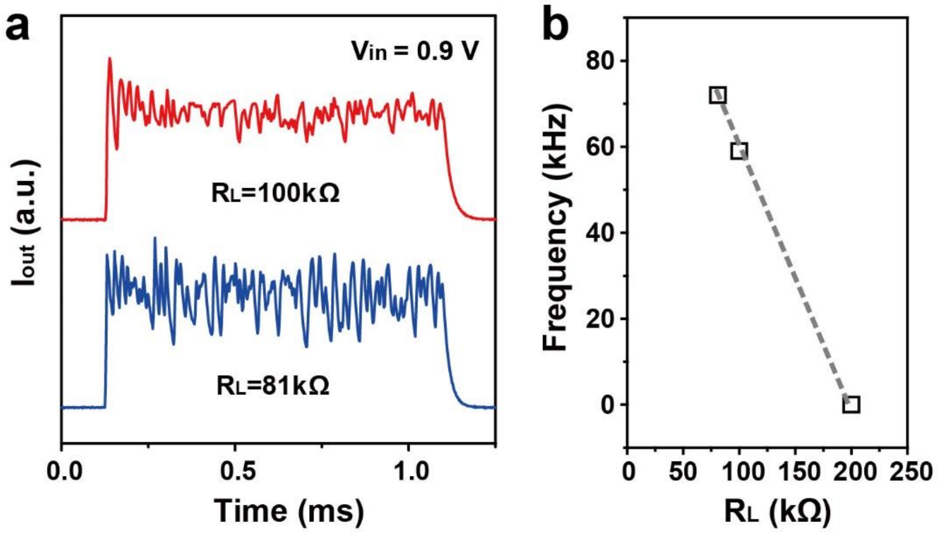

Figure S5. Output oscillation frequency of TS oscillatory neuron responds to the input synapse weights. (a) Oscillation current responds to input pulse of 0.9 V when TS series with R_L= 81 kΩ and 100 kΩ, respectively. (b) The relation between output frequency and series resistance.

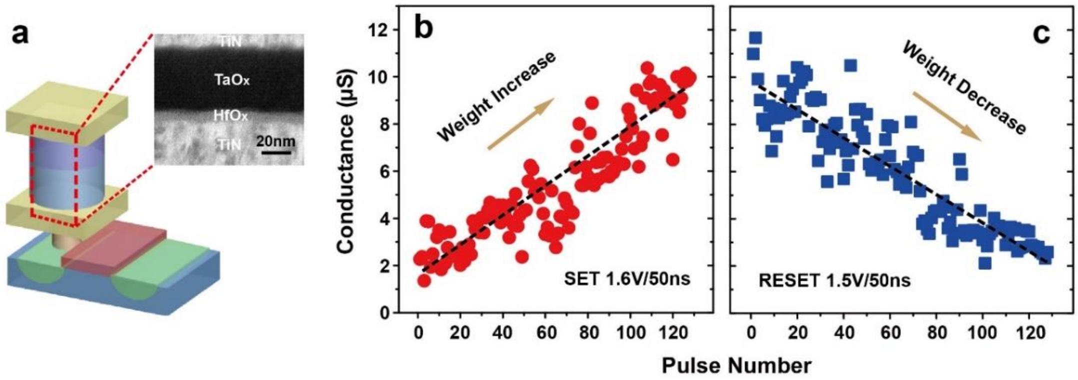

Figure S6. Dynamics of RS synapse. (a) Schematic illustration of a single one-transistor-one-resistor (1T1R) cell structure, and the cross-sectional scanning transmission electron microscopy (STEM) image of RS stacks (TiN/HfO_x/TaO_x/TiN). (b) Continuous conductance tuning performance under pulse train condition during SET operation (1.6 V, 50 ns). (c) Continuous conductance tuning performance during RESET operation (-1.5 V, 50 ns).

The design and fabrication of RS synapses:

1Kb 1T1R array with TiN/TaO_x/HfO_x/TiN stack (size: 1.2 × 1.2 μm^2)[1] is used as RS synapses, and the structure is schematically illustrated in Figure S6a. The transistors and interconnet wires are firstly fabricated in a CMOS foundary. The HfO_x layer is deposited on the TiN bottom electrode by ALD acting as a switching layer (SWL), and the TaO_x layer is sputtered serving as an electro-thermal modulation layer (ETML), which works not only as a thermal enhanced layer but also promotes to form uniform Vo distribution. And then, the top electrode of Al/TiN is sputtered. Finally, the top Al pad is patterned by dry etching with Cl_2/BCl_3 plasma. Remarkably, the introducing of TaO_x ETML with a suitable resistivity by optimized oxygen composition can contribute to improving the linearity of filamentary analog RS synapse. Clearly, a good linearity is observed on the device for both SET and RESET with the optimized analog RS synapse (Resistivity of ETML: ~30.4mΩ·cm). Moreover, the ETML/HfOx based memristor shows low operating current and large dynamic tuning window of analog switching (Figure S6a,b), which is crucial for scaling up the size of neural network.

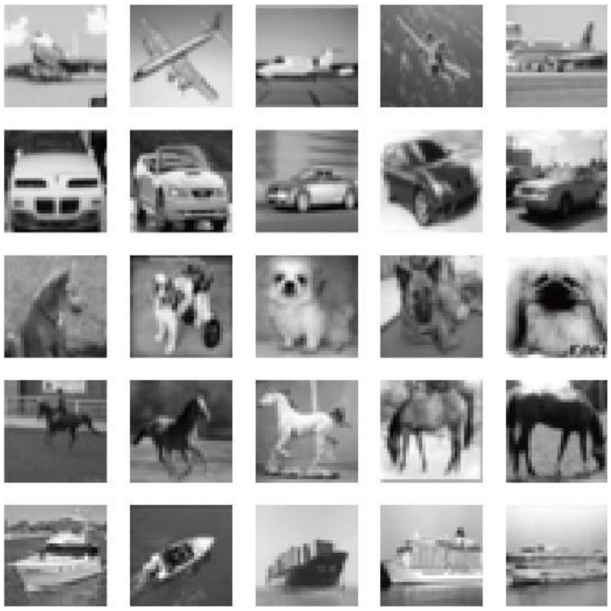

Figure S7. Image examples from the subset of grayscale CIFAR-10 used in this study. Five classes of images are chosen: airplane/automobile/dog/horse/ship.

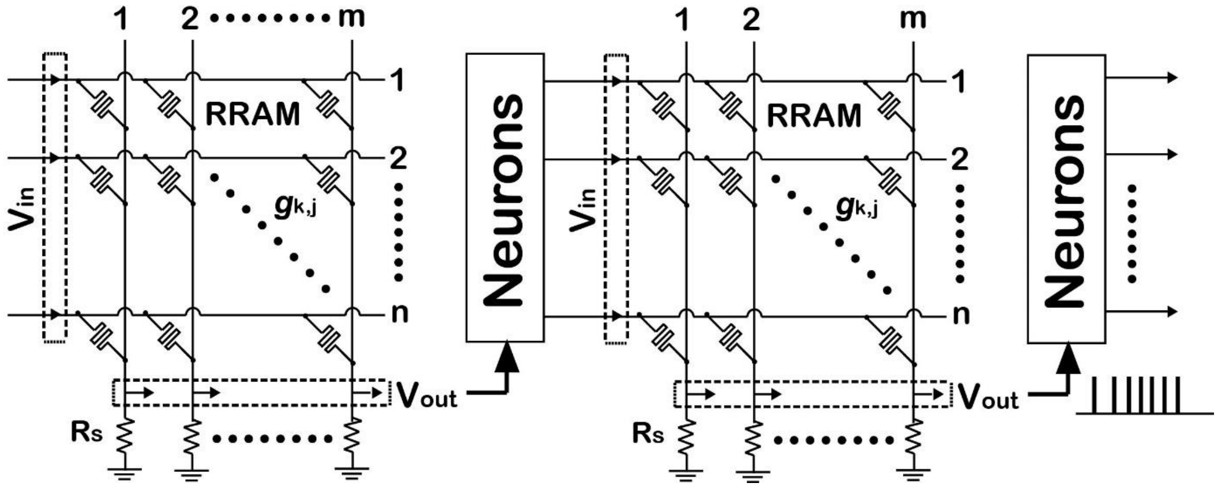

Figure S8. Schematic illustration of memristor-based spiking neural network system structure including RS array synapses and TS oscillatory neurons.

Table S1. Comparison of oscillatory neurons based on different materials.

Material	Voltage	Power	Endurance	Reference
**PCM**	5.3 V	4.3 μW	-	[2]
**TaO_x**	6 V	200 μW	-	[3]
**NbO_2**	1.7 V	10 μW	-	[4]
**VO_2**	0.8 V	23.75 μW	$> 2.5\times10^9$	[5]
**Ag/HfO_2**	0.6 V	1.8 μW	$> 10^8$	This work

Table S2. The performance comparison of various metal-filamentary threshold switching memristors.

**Device Structure**	**I_{on}**	**I_{off}**	**On/off ratio**	**Ref.**
$Ag/TiO_2/Pt$	10μA (I_{cc})	~1pA	10^7	[6]
$Pt/HfO_2/Cu:HfO_2/Cu$	10μA (I_{cc})	~1pA	10^7	[7]
$Pt/Cu_2O/Ag:Cu_2O/Cu_2O/Pt$	1μA	~1nA	10^3	[8]
Ag/a-Si:H/Pt	10μA (I_{cc})	~1pA	10^7	[9]
$Cu/SiO_2/Pt$	500μA (I_{cc})	~10pA	10^7	[10]
$Ag/ZrO_2/Pt$	1mA (I_{cc})	~0.1nA	10^7	[11]
$AgTe/TiN/TiO_2/Pt$	100μA (I_{cc})	~1pA	10^8	[12]
$W/Cu_2S/W$	10μA (I_{cc})	~100pA	10^5	[13]
Ag/HfO_2/p-Si	100μA (I_{cc})	~10pA	10^7	[14]
$Ag/SiO_x/C$	50μA (I_{cc})	~1pA	$>10^7$	[15]
$Pd/Ag/HfO_x/Ag/Pd$	100μA (I_{cc})	~1pA	10^8	[16]
Pt/Ag:ZnO/Pt	100μA (I_{cc})	~0.1pA	$>10^9$	[17]
$Ag/Gr/SiO_x/Pt$	500μA (I_{cc})	<1pA	$5x10^8$	[18]
Pt/Ag nanodots/HfO_2/Pt	>1mA	<1pA	$>10^9$	[19]

Reference

[1] W.Wu, H.Wu, B. Gao, P. Yao, X. Zhang, X. Peng, S. Yu, H. Qian, presented at *2018 Symp. VLSI Technology (VLSIT),* Honolulu, HI, USA,18-22 June **2018**.

[2] T. Tuma, A. Pantazi, M. Le Gallo, A. Sebastian, E. Eleftheriou, *Nat. Nanotechnol.* **2016**, *11*, 693.

[3] A. A. Sharma, Y. Li, M. Skowronski, J. A. Bain, J. A. Weldon, *IEEE Trans. Electron Devices* **2015**, *62*, 3857.

[4] M. D. Pickett, G. Medeiros-Ribeiro, R. S. Williams, *Nat. Mater.* **2013**, *12*, 114.

[5] J. Lin, A. Annadi, S. Sonde, C. Chen, L. Stan, K.V.L.V. Achari, S. Ramanathan, S. Guha, presented at *2016 IEEE Int. Electron Devices Meet. (IEDM),* San Francisco, CA, USA, 3-7 Dec. **2016**.

[6] J. Song, J. Woo, A. Prakash, D. Lee, H. Hwang, *IEEE Electron Device Lett.* **2015**, 36, 681.

[7] Q. Luo, X. Xu, H. Liu, H. Lv, T. Gong, S. Long, Q. Liu, H. Sun, W. Banerjee, L. Li, N. Lu, M. Liu, presented at *2015 IEEE Int. Electron Devices Meet. (IEDM)*, Washington, DC, USA, 7-9 Dec., **2015**.

[8] J. Song, A. Prakash, D. Lee, J. Woo, E. Cha, S. Lee, H. Hwang, *Appl. Phys. Lett.* **2015**, 107, 113504.

[9] J. Yoo, J. Woo, J. Song, H. Hwang, *AIP Adv.* **2015**, 5, 127221.

[10] W. Chen, H. J. Barnaby, M. N. Kozicki, *IEEE Electron Device Lett.* **2016**, 37, 580.

[11] G. Du, C. Wang, H. Li, Q. Mao, Z. Ji, *AIP Adv.* **2016**, 6, 085316.

[12] J. Song, J. Park, K. Moon, J. Woo, S. Lim, J. Yoo, D. Lee, H. Hwang, presented at *2016 IEEE Int. Electron Devices Meet. (IEDM)*, San Francisco, CA, USA, 3-7 Dec., **2016**.

[13] S. Lim, J. Yoo, J. Song, J. Woo, J. Park, H. Hwang, presented at *2016 IEEE Int. Electron Devices Meet. (IEDM)*, San Francisco, CA, USA, 3-7 Dec., **2016**.

[14] N. Shukla, B. Grisafe, R. K. Ghosh, N. Jao, A. Aziz, J. Frougier, M. Jerry, S. Sonde, S. Rouvimov, T. Orlova, S. Gupta, S. Datta, presented at *2016 IEEE Int. Electron Devices*

Meet. (IEDM), San Francisco, CA, USA, 3-7 Dec., **2016**.

[15] A. Bricalli, E. Ambrosi, M. Laudato, M. Maestro, R. Rodriguez, D. Ielmini, presented at *2016 IEEE Int. Electron Devices Meet. (IEDM)*, San Francisco, CA, USA, 3-7 Dec., **2016**.

[16] R. Midya, Z. Wang, J. Zhang, S. E. Savel'ev, C. Li, M. Rao, M. H. Jang, S. Joshi, H. Jiang, P. Lin, K. Norris, N. Ge, Q. Wu, M. Barnell, Z. Li, H. L. Xin, R. S. Williams, Q. Xia, J. J. Yang, *Adv. Mater.* **2017**, 29, 1604457.

[17] U.-B. Han, D. Lee, J.-S. Lee, *NPG Asia Mater.* **2017**, 9, e351.

[18] X. Zhao, J. Ma, X. Xiao, Q. Liu, L. Shao, D. Chen, S. Liu, J. Niu, X. Zhang, Y. Wang, R. Cao, W. Wang, Z. Di, H. Lv, S. Long, M. Liu, *Adv. Mater.* **2018**, 1705193.

[19] Q. Hua, H. Wu, B. Gao, M. Zhao, Y. Li, X. Li, X. Hou, M. F. Chang, P. Zhou, H. Qian, *Adv. Sci.* **2019**, 1900024.
